# The healthy moms and babies app to prevent postpartum depression: analysis of user-profiles and dropout cases

**DOI:** 10.3389/fpubh.2023.1186963

**Published:** 2023-09-15

**Authors:** María F. Rodriguez-Muñoz, Katina Kovacheva, Helena S. García-López, Huynh-Nhu Le, Natalia Ruiz, Maria Eugenia Olivares, Nuria Izquierdo Mendez, Pluvio Coronado

**Affiliations:** ^1^School of Psychology, Universidad Nacional de Educación a Distancia, Madrid, Spain; ^2^Psychology and Social Sciences, University of Maryland Global Campus (Europe Site), Adelphi, MD, United States; ^3^Department of Psychological and Brain Sciences, George Washington University, Washington, DC, United States; ^4^Faculty of Medicine, Department of Gynecology and Obstetrics, Instituto de Salud de la Mujer José Botella Llusiá, Hospital Clínico San Carlos, Universidad Complutense de Madrid, Madrid, Spain

**Keywords:** perinatal depression, m-health, mobile app, healthcare, dropout, prevention

## Abstract

**Introduction:**

Perinatal depression affects mothers, babies and society. Preventive interventions are needed, but face barriers to access. E-health interventions could be an effective and accessible option. To date, few studies have attempted to understand the use of mobile health (m-health) applications and why they are not more widely used. This study aims to understand the demographic characteristics of enrolled participants and examine dropout patterns through the Healthy Moms and Babies app.

**Methods:**

A longitudinal study was conducted with a sample of 511 women recruited between 2020 and 2022. Data were collected from the app, including sociodemographic information, the participant’s progress through the modules of the app, and the permissions granted to use the app.

**Results:**

Out of the 511 women who completed the initial form to initiate participation, 279 downloaded the app and completed the evaluation. Results indicated that granting permission to be notified about the module’s availability is related to an increase in the use of the first modules.

**Conclusion:**

This study shows the importance of establishing follow-ups in the use of mobile apps during the perinatal period.

## 1. Introduction

Depression is the most common psychological disorder during the perinatal period (1), making it a major public health problem, with a global prevalence around 11.9% (2). This prevalence has increased in recent years due to COVID-19, reaching a prevalence ranging from 22 to 31% (3, 4).

PD is characterized by a non-psychotic depressive episode occurring during pregnancy or in the first year following childbirth (5). Several related risk factors have been highlighted in the scientific literature, such as age, primiparity, not having a partner (6), educational level (7), anxiety or severe levels of stress (8), gender violence (9), and poor social support or mental health history among others (4, 6). Cases of underdiagnosis or inadequate treatment for women with PD can lead to negative consequences for both mothers and babies (5). In the most severe cases, it can even lead to suicide (10).

Research has found high rates of technological adoption, some data show that, among women in the perinatal period, 94% report using the internet, 90% report using email, and 50% report using social networks (e.g., Twitter) (11). Within the new technologies, smartphone stands out among the systems used by perinatal women (12).

Mobile technologies are bringing about a significant transformation in public health (12) because they offer the possibility of being used as a coordination tool between patients and healthcare professionals. An increasing number of studies conclude that the use of a mobile health (mHealth) is helpful, given the high frequency of use and its effectiveness in improving and maintaining health (13). Among the types of mHealth, it is worth highlighting health applications, which have high potential as a cost-effective and accessible intervention (13). However, the explored literature exposes a high dropout rate in these systems, estimated at around 43% of the users (14).

Understanding the use of mHealth applications is an increasingly relevant research topic, which is aligned with the dynamic lifestyle of this century. Technology adoption models attempt to explain the decision-making processes for mHealth use, highlighting consumer expectations regarding ease of use and the benefits that can be obtained, as the main predictors of the intent of use (15). Much of this research has focused on applications developed and accessible in English, yet Spanish-speaking populations are numerous. Spain leads the world ranking in penetration with 88% of mobile users, there are 27.7 million active users who have an average of 17.8 apps installed on their smartphones (16).

However, despite the widespread use of applications in this context, there is limited research regarding the use of apps, in general, but especially related to mental health, suggesting a gap in research and highlighting the need for further research in this field (17). To date and to the best of our knowledge, the app “Mamás y Bebés Saludables” [Healthy Moms and Babies] (18) is the only app in Spanish that is currently in use in the field of perinatal care and aims to provide psychoeducation and prevention of PD.

To the best of our knowledge, there is a paucity of research related to the usability of mental health applications during the perinatal period. Therefore, the purpose of this study is to examine the demographic characteristics of registered users of the “Healthy Moms and Babies” mobile app, as well as to analyze the attrition rate and understand the patterns associated with attrition. It is intended that the results of this study will provide a better understanding of app usage behaviors, with the goal of promoting more effective adoption and use of the app.

## 2. Methods

### 2.1. Design

The study data were collected from the Healthy Moms and Babies app, whose study and intervention protocol is published (18). It is a randomized controlled design, but it should be noted that, for this analysis, we took into consideration users who did not complete the assessment and, therefore, did not belong to the intervention group. However, we decided to include this sample in the analysis to better understand the dropout process and its possible implications on the results.

### 2.2. Participants

The sample included 511 pregnant women undergoing obstetric follow-up at the Hospital Clínico San Carlos in Madrid (Spain), recruited between 2020 and 2022 in an urban setting, and also women recruited from social networking sites all over the country.

Inclusion criteria were: (i) currently pregnant (participants could join the study at any time during their pregnancy); (ii) 18 years of age or older; (iii) Spanish fluency; (iv) possess a smartphone and basic knowledge of usage; and (v) providing consent to participate in the study.

### 2.3. Procedure

The study was approved by the Ethics Committee of San Carlos Clinics Hospital (IRB Number: 19/184-E), in accordance with the principles expressed in the Declaration of Helsinki. The study was voluntary, and the confidentiality of all information collected was guaranteed.

Users were recruited through social networks (Twitter and LinkedIn), from the web page of the Mamás y Bebés project,^1^ and through the obstetricians and midwives of the San Carlos Clinic Hospital in Madrid, Spain.

Access to the initial web form was available through a QR code for users to scan with a cell phone. The initial form asked for the informed consent of the participants and collected contact information and sociodemographic data (age, gestational week, place of origin and residence, reference hospital, studies completed, employment status, marital status, and a number of pregnancies, cesarean sections and/or previous abortions). Lastly, participants received an e-mail thanking them for their participation, which also contained instructions for downloading the app and personal access codes.

The subjects accessed the application through a code without providing any personal information, thus ensuring an anonymized database. The server, in turn, belonged to the National University of Distance Education (UNED), so that privacy was guaranteed.

### 2.4. App information

The primary objective of the Healthy Moms and Babies App was the prevention of depressive symptomatology during the postpartum period. The secondary objective was to prevent anxiety symptoms after childbirth. As a third aim, the application raises awareness among pregnant women about the importance of taking care of their mental health and psychological well-being during the perinatal period (18). The intervention was developed in 11 modules. All the modules have the same structure (introductory video, psychoeducational content, a practical exercise, guidelines, and a final motivational message). The App’s content was based on the Mothers and Babies Course for preventing postpartum depression. This intervention has demonstrated strong empirical evidence based on the principle of cognitive behavioral therapy (CBT), and has been considered a gold standard (19). CBT is one of the most studied interventions for the treatment and prevention of PD (18). Mothers and Babies Course comes from the United States and has been adapted to the Spanish cultural context, recruiting pregnant women at high risk of PE in their first trimester in an obstetrics clinic in two urban hospitals in Spain (20). The application is interactive and allows users to answer questionnaires, do exercises, watch videos or listen to audios. Also, the application asks users for permission to receive reminder information.

### 2.5. Instruments

The Patient Health Questionnaire (PHQ-9) (21) was used to assess depressive symptoms before to start and at the end of the modules. It consists of nine items with a Likert-type scale ranging from 0 (never) to 3 (almost every day) in the last 2 weeks. The total score is obtained by the direct sum of the responses for each of the items. The higher scores indicate high severity of depressive symptoms (range 0–27), with five different categories: (a) Minimal depression (scores 0–4); (b) Mild depression (5–9); (c) Moderate depression (10–14); (d) Moderately severe depression (15–19); and (e) Severe depression (20–27). This questionnaire has shown good psychometric properties in a Spanish-speaking population (22). For the purpose of this study, and based on the PHQ-9 score, called the $value, the Plesk software distributed participants according to their level of symptomatology. Eligible patients (PHQ-9 score between 5 and 19) were randomly assigned to the intervention and control groups according to the standard PHP function [mt_rand()] automatically generated by a computer. An independent researcher programed the mathematical algorithm that performs the randomization. Those women with a score above 19 were recommended to seek help from a health professional.

### 2.6. Statistical analysis

The statistical analysis was carried out using the SPSS Statistics program for Windows (version 24), setting the confidence level at 95% and the significance levels at 5% (*p* < 0.05). The differences regarding the categorical variables in each of the groups were analyzed, contingency tables were made and the Pearson Chi-Square statistic was applied. For the variables with mean scores obtained for each of the scales, comparisons of the means were made using the Student’s t-test for independent samples was used. In addition, Cramer’s V effect size index and Cohen’s d were calculated according to the following interpretation of the scale: 0–0.19, insignificant; 0.20–0.49, small; 0.50–0.79, medium; more than 0.80, high (23).

## 3. Results

**Figure 1:**
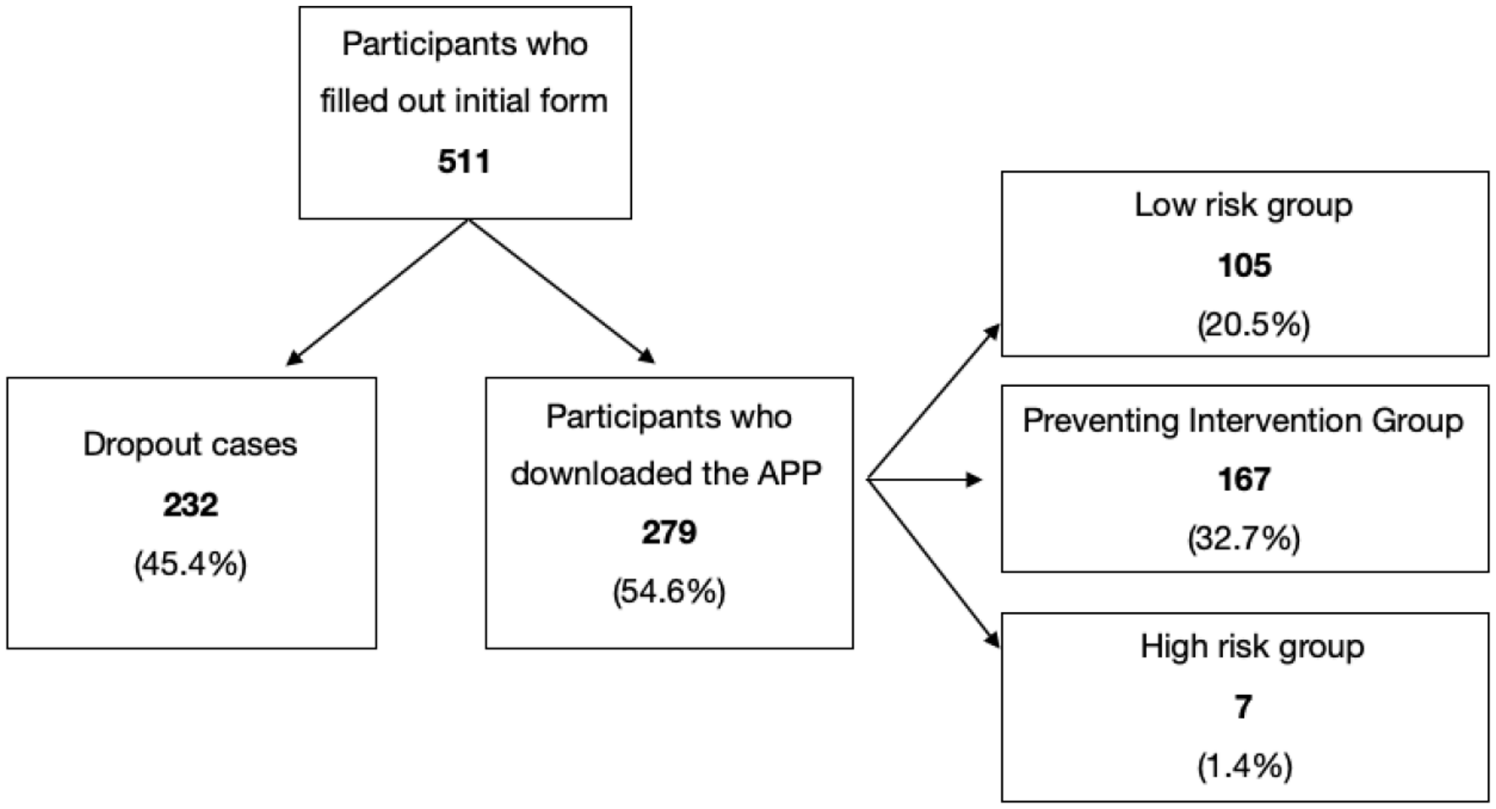
Distribution of the sample. Prepared by the authors.

As shown in Figure 1, out of the 511 female users who completed the initial form, 279 (54.6%) downloaded the mobile application and completed the assessment. Data from the initial assessment period indicated that 105 women (20.5%) obtained a low PHQ-9 score (0–4), another 167 participants (32.7%) obtained a score indicating depressive symptomatology (5–19), and 7 women (1.4%) were considered in the severe depression range (20–27) and who were referred to a mental health professional.

### 3.1. Participant characteristics

Table 1 shows the main sociodemographic characteristics of the participants in relation to the following: the entire sample, participants who did not download the application (dropouts), and participants who continued using the app (initiators). For all participants, the mean for age was 34.34 (SD = 4.65) years; the majority reported living with a partner (90.1%), having a college-level education (74.2%), and employed (71.8%). Most participants did not previously have a cesarean section (93.2%); for slightly more than half of the participants this was their first pregnancy (58.1%), and they had not had previous abortions (67.1%). Approximately half of the sample started the application, specifically 279 of the 511, and both groups (dropouts vs. initiators) had similar sociodemographic characteristics, except for gestation. The mean of number of gestational weeks was lower in those women who initiated the application compared to those who dropped out and did not download the App (*p* = 0.05; *d* = 0.181).

**Table 1:** Participant characteristics.

Sociodemographics variables	All participants *N* = 511 *n* (%) or Mean (SD)	Participants dropped out of App *n* = 232 *n* (%) or Mean (SD)	Participants initiated App *n* = 279 *n* (%) or Mean (SD)
Age	34.34 (4.65)	34.55 (4.74)	34.17 (4.58)
Gestation week	18.40 (9.88)	19.37 (9.92)*	17.58 (9.79)*
Level of education			
Elementary school	25 (5%)	13 (5.7%)	12 (4.3%)
High school	105 (20.8)	54 (23.7%)	51 (18.5%)
College level	374 (74.2%)	161 (70.6%)	213 (77.2%)
Marital status			
Married/living with a partner	457 (90.1%)	203 (88.6%)	254 (91.4%)
Single	46 (9.1%)	25 (10.9%)	21 (7.6%)
Divorced/separated	4 (0.8%)	1 (0.4%)	3 (1.1%)
Employment situation			
Active	363 (71.7%)	164 (71.9%)	199 (71.6%)
Unemployed	85 (16.7%)	37 (16.2%)	48 (17.3%)
Homemaker	36 (7.1%)	20 (8.8%)	16 (5.8%)
Disabled	22 (4.3%)	7 (3.1%)	15 (5.4%)
Number of previous pregnancies	0.67 (0.97)	0.74 (1.02)	0.62 (0.94)
Number of previous C-section	0.07 (0.28)	0.08 (0.30)	0.07 (0.26)
Number of miscarriage	0.48 (0.81)	0.48 (0.83)	0.47 (0.79)

*p = 0.05.

### 3.2. Descriptive and comparative analysis

Out of the 279 participants who continued using the application, only 42 participants (15.1%) allowed the application to notify them of the subsequent available modules, compared to 237 (84.9%) who did not.

Table 2 presents an in-depth comparison of the data obtained from women who give permission to the application to notify them of the available modules regarding the completion of each of them. Significant differences exist in the completion of the first 4 modules. Women who enabled app notifications for the availability of the modules had a higher percentage of completion of module 1 (33.3% vs. 2.6%) compared with the rest of the modules’ completion: module 2 (25% vs. 11.1%), module 3 (20.4% vs. 15.7%), and module 4 (18.5% vs. 17.6%).

**Table 2:** Descriptive and comparative analysis between modules “completed” and “permissions for the notification of module availability.”

Module		Participants who gave permission to receive notice of availability *N* = 39 n (%)	Participants who did not give permission to receive notice of availability *N* = 69 n (%)	*P*	Effect Size
1. “You are a mum”	Completed	36 (33.3%)	40 (37%)	<0.001	0.361
	Not completed	3 (2.8%)	29 (26.9%)		
2. “Psychoeducation”	Completed	27 (25%)	32 (29.6%)	0.022	0.220
	Not completed	12 (11.1%)	37 (34.3%)		
3. “Feel better”	Completed	22 (20.4%)	24 (22.2%)	0.029	0.210
	Not completed	17 (15.7%)	45 (41.7%)		
4. “Thoughts”	Completed	20 (18.5%)	22 (20.4%)	0.047	0.191
	Not completed	19 (17.6%)	47 (43.5%)		
5. “Enjoy the day”	Completed	16 (14.8%)	20 (18.5%)	0.202	0.123
	Not completed	23 (21.3%)	49 (45.4%)		
6. “Relax”	Completed	13 (12%)	13 (12%)	0.091	0.163
	Not completed	26 (24.1%)	56 (51.9%)		
7. “Your body”	Completed	10 (9.3%)	10 (9.3%)	0.152	0.138
	Not completed	29 (26.9%)	59 (54.6%)		
8. “Find a solution”	Completed	10 (9.3%)	10 (9.3%)	0.152	0.138
	Not completed	29 (26.9%)	59 (54.6%)		
9. “Social support”	Completed	9 (8.3%)	9 (8.3%)	0.179	0.129
	Not completed	30 (27.8%)	60 (55.6%)		
10. “Goodbye myths”	Completed	6 (5.6%)	8 (7.4%)	0.573	0.054
	Not completed	33 (30.6%)	61 (56.5%)		
11. “Your baby”	Completed	6 (5.6%)	8 (7.4%)	0.573	0.054
	Not completed	33 (30.6%)	61 (56.5%)		

### 3.3. Frequency

Table 3 shows the frequency of completion of the modules, which also indicates the dropout rate. Module 1 was completed by 70.4%, with the highest dropout rate occurring between modules 1 and 2. In module 2 the dropout rate decreased to 54.6%, followed by the second highest dropout rate between modules 2 and 3, where the completion rate is 42.6%. The following modules 4 and 5 still show a loss of users, but between modules 5 and 6 there is the third highest dropout rate, with a completion rate for module 6 of 24.1%. From module 6 onwards, there is the lowest loss of users with a completion rate in module 11 of 13%.

**Table 3:** Frequency of module completion (*N* = 108).

*M*1	*M*2	*M*3	*M*4	*M*5	*M*6	*M*7	*M*8	*M*9	*M*10	*M*11
76	59	46	42	36	26	20	20	18	14	14
(70.4%)	(54.6%)	(42.6%)	(38.9%)	(33.3%)	(24.1%)	(18.5%)	(18.5%)	(16.7%)	(13%)	(13%)

## 4. Discussion

Mobile technologies can be transformative for public health thanks to the “always connected” trend by providing an unprecedented opportunity to facilitate change in users’ health behaviors (24). The format of web pages or applications can be used repeatedly by an unlimited number of people, without any spatiotemporal limitation (25). This creates the need to explore the possibilities of this channel of intervention and also discover how to promote its adoption and use, which is the overall goal of this study.

Some studies indicate that women are more likely to use mobile health apps than others (24). Particularly, pregnant women show a greater interest in using technology for accessing information related to pregnancy and postpartum care (26). Indeed, our study found that the earlier gestational period is related to more engagement with the application. It is possible that women have less information and more time during the early part of pregnancy to view technology as more beneficial and useful.

Regarding the adoption and use of apps, our study shows a relatively high dropout rate, which is consistent with the existing literature (14). Understanding the reasons for dropout is essential to improve the effectiveness of these apps. Effortless navigation and ease of use have been identified as critical factors influencing intention to use (27). Simplifying the process of accessing the application could help increase downloads and engagement.

In addition, our research underscores the importance of timely and personalized notifications as effective motivators for sustained app use. Regular reminders and motivational messages can keep users engaged, especially in the early stages of app usage (28). This is a common strategy to encourage usage, especially in the early stages, as users are more motivated to receive suggestions.As users become more familiar with the app and become regular consumers, they require higher quality and more personalized notifications to achieve greater responsiveness (29). The phase with the highest rate of abandonment in our sample is between the first and second module, because this is when the user has the opportunity to evaluate the usefulness and ease of use of the application. Therefore, a user-friendly interface with intuitive, accessible, attractive and personalized navigation are important factors to reduce the abandonment rate. Other studies also highlight the importance of setting clear goals and generating intrinsic motivation, and a game-like presentation can contribute to the level of participation in the application (30).

Our study provides valuable insights for improving the adoption and use of mobile health applications. By understanding user preferences, motivations, and behaviors, we can design more effective and engaging applications to promote positive health behaviors in the target population.

### 4.1. Study limitations

This study has limitations that could be addressed in future research. It would be interesting to use behavioral data recorded on mobile devices to analyze usage patterns, circadian rhythms, and the difference between days of mHealth app use, to understand how and when they use them. These types of studies around the understanding of app usage behavior may boost usage among pregnant women at risk of developing perinatal depression, as part of their prenatal care. This will benefit pregnant women by contributing to the prevention of depression, the realization of an early diagnosis, and fostering a space to address the importance of mental health in medical consultation. Also add the limitation of the number of people who participated in the study. Data collection took place during the coronavirus season, which possibly made it difficult to obtain the sample due to the abundance of other studies being promoted on social networks in that period.

## 5. Conclusion

Our study provides valuable theoretical and practical information for public health. We can mention that although the low participation and dropout rate might suggest some disinterest in the use of the application in this specific sample, it is essential to consider the aspects observed in this study to identify the possible barriers and challenges that women face in relation to the use of mental health applications during the perinatal period. First, we highlight the importance of providing access to such applications from the first medical consultations during the prenatal period. Second, we identify the need to improve accessibility and create user-friendly health platforms to enhance the user experience. Finally, we show that reminder notifications have a positive impact on engagement with the application and help develop usage habits. Therefore, offering personalized follow-up services can keep users more engaged with the app. These findings will not only help us improve our own app, but we also hope that they will be considered by mobile health app developers when creating future health projects.

## Data availability statement

The raw data supporting the conclusions of this article will be made available by the authors, without undue reservation.

## Ethics statement

The studies involving humans were approved by the Ethics Committee San Carlos Clinic Hospital. The studies were conducted in accordance with the local legislation and institutional requirements. The participants provided their written informed consent to participate in this study.

## Author contributions

MR-M: study conception and design. NR: data collection. KK: analysis and interpretation of results. KK, MR-M, and HG-L: draft manuscript. NI, MO, and PC: preparation. All authors reviewed the results and approved the final version of the manuscript.

## Conflict of interest

The authors declare that the research was conducted in the absence of any commercial or financial relationships that could be construed as a potential conflict of interest.

## Publisher’s note

All claims expressed in this article are solely those of the authors and do not necessarily represent those of their affiliated organizations, or those of the publisher, the editors and the reviewers. Any product that may be evaluated in this article, or claim that may be made by its manufacturer, is not guaranteed or endorsed by the publisher.

## Footnotes

^1^ https://blogs.uned.es/mbc/
